# Application of propensity scores to explore the effect of public reporting of medicine use information on rational drug use in China: a quasi-experimental design

**DOI:** 10.1186/s12913-014-0492-6

**Published:** 2014-11-11

**Authors:** Xiaopeng Zhang, Lijun Wang, Xinping Zhang

**Affiliations:** School of Medicine and Health Management, Tongji Medical College, HuaZhong University of Science and Technology, Wuhan, Hubei Province China

**Keywords:** Quasi-experimental design, Propensity score, Township hospitals, Transparency, Rational drug use, China

## Abstract

**Background:**

Transparency has become a hottest topic and a growing movement in the health care system worldwide. This study used a quasi-experimental design method to explore whether public reporting of medicine use information can improve rational drug use.

**Methods:**

20 township hospitals and 274 doctors of City Y in Hubei Province, China were divided into the intervention and control groups on the basis of their characteristics. In the intervention group, the values and rankings of the average expenditure per prescription, percentage of prescriptions requiring antibiotics and percentage of prescriptions requiring injections of each hospital and doctor were publicly released to patients and doctors in an appropriate format monthly. Data were gathered both four months before and after the intervention. Propensity score matching (PSM) was used to minimize the observed covariate (gender, age, experience, education level, title, and monthly income) differences in the doctors’ characteristics. 108 pairs of doctors were obtained after PSM. Chi-square test and t-test were employed to explore the effect of public reporting of medicine use information on rational drug use. The study was approved by the Committee of Tongji Medical College, Hua Zhong University of Science and Technology (IORG No: IORG0003571).

**Results:**

In baseline, the average expenditure per prescription of the 274 doctors was 42.82 RMB yuan (USD 6.97), the percentage of prescriptions requiring antibiotics was 63.00%, and the percentage of prescriptions requiring injections was 70.79%, all higher than the average of Hubei Province and the standard recommended by WHO. Before the intervention all the three indicators were all comparable (p > 0.05), whereas after the intervention, a significant difference (p < 0.05) was found for the percentage of prescriptions requiring injections between the intervention (64.66%) and control groups (70.52%).

**Conclusions:**

Irrational drug use remains a policy issue in township hospitals in the study area. We demonstrated that publicly reporting medicine use information could decrease the percentage of prescriptions requiring injections in township hospitals in China, but this effect was not observed on prescription costs and antibiotics use. Analyses of the mechanism and long-term effect of public reporting of medicine use information are recommended for further studies.

## Background

Transparency has become one of the hottest topics in the health care system worldwide. The Institute of Medicine defined health care transparency as making health care available to the public in a reliable and understandable manner, with a primary focus on the reporting of information and processes [1]. In this study, we discuss public reporting of information. According to the literature, health care transparency can: (a) ensure that consumers make informed health care choices; (b) increase trust in the patient–physician relationship and health care systems; (c) improve performance (quality, safety, and efficiency) throughout the health care system because of competition and/or availability of clinical benchmarks [2,3]. Berwick proposed that public reporting can improve performance via two pathways, the selection and the change pathways [4]. In the selection pathway, patients or their intermediaries compare publicly released performance data and reward the better-performing providers by “selecting” (rewarding, recognizing, or paying) that provider. In the change (or quality improvement) pathway, performance data help providers identify areas in which they underperform and improve their performance accordingly [4-6].

In fact, public reporting of quality information is a growing movement in many countries. In US health care system, almost every state has quality reporting programs for hospitals, and the Center for Medicare and Medicaid Services has initiated public disclosure programs for hospitals, managed care plans, nursing homes, and home health agencies [7]. In South Korea, a national insurance review agency has been publicly releasing information about antibiotics use rates among health care organizations since 2006 [8]. Some other countries have also attempted to develop and collect quality measures that capture consumer experience with health care systems [9]. In 2009, the Chinese government established the document “Views on Deepening the Reform of Medical Care System”, which points out the importance of increasing drug regulatory transparency. Certain links in the drug-use segment, such as antibiotics use, prescription comment, drug expenditure reimbursement, and so on, were very important parts of the regulation and were also the most urgent parts that have to be transparent. Many local governments had done certain attempts, but no results have been published yet.

As public reporting rapidly expands, several studies have examined the effects of information disclosure on quality in hospitals or nursing home markets. A review synthesized the evidence for using publicly reported performance data to improve quality, but found mixed results [5]. After that, other studies have been published. Won Mo Jang [10] found that repeated public releases on Caesarean section rates had no significant effect on reducing Caesarean section rates. In addition, Bundorf [11] evaluated a reporting program for Medicare HMOs and found that public reporting has no significant positive effect on quality. However, Rachel [12] found that public reporting in post-acute care setting had mixed effects in areas without public reporting, which improved in high-ranking facilities but worsened in low-ranking facilities. Jung also reported that release of quality information had a significant positive effect on the quality in HMO markets during the earlier years of the voluntary disclosure program. Hence, the effect, if any, these activities have on quality improvement is still unclear.

In view of methodology, few studies used quasi-experimental method or had control groups. In Bundorf’s study, although a comparison group was set, a new policy was implemented during the study period. Jung’s study also had a control group, but it involved secondary data analysis in which the confounding factor was not matched. Hibbard [13] used a quasi-experimental method and set control group, but the control group was not randomly selected. Hence, a high-quality experimental or quasi-experimental design is needed to determine whether public reporting of information has an effect on quality improvement.

In our study, a quasi-experimental design method was used to explore whether public reporting of medicine use information can improve the doctor’s prescribing practice in City Y, Hubei Province of China. The results can enrich the evidence of health care transparency on quality and provide references for China and other countries to promote health care transparency and rational drug use.

## Methods

### Study design and setting

A quasi-experimental design was used, and the intervention was implemented in November 2013. Our study was conducted in City Y, a city located in the central part of Hubei Province, China. Twenty township hospitals (total 22) were selected as intervention group or control group. First, Qianjiang had a total of 22 township hospitals. On the basis of hospital characteristics, including service population, number of doctors and outpatients, and revenue training times on rational drug use, the 22 hospitals were numbered from 1 to 22 using technique for order preference by similarity to an ideal solution (TOPSIS) method, and the first 20 hospitals were chosen as the sample. Second, starting with the first hospital, the two hospitals that had the nearest comprehensive evaluation index were chosen as a pair. Hence, a total of 10 pairs were formed. Third, coin tossing method was used to divide the two hospitals in each pair into the intervention group and the control group. In the end, each group had 10 hospitals.

### The intervention

Medicine use information was made public to the patients and doctors in the intervention group, including the values and rankings of average expenditure per prescription, percentage of prescriptions requiring antibiotics, and percentage of prescriptions requiring injections of each hospital and each doctor who had the privilege of prescribing medications. First, the medicine use information was calculated using the data from the information system of the Health Bureau of City Y. To ensure the correctness of the calculations, six researchers participated in this work and checked one another. Second, the information was printed in the appropriate format in A3/A4 paper. Third, the researchers posted the A3 papers on the bulletin board in the hall of hospitals and distributed the A4 papers to each doctor and director of the hospitals monthly.

According to Berwick’s theory, the intervention’s effects might come from three ways: (1) patient’s rewarding the better-prescribing doctors after seeing the medicine use information; (2) pressure from the director of each hospital to improve their ranking; (3) doctors’ effort to improve prescription behaviors to gain more patients or better reputation. However, in this paper, we do not distinguish the specific way from which the effect comes.

### Data sources

First, in the baseline, the characteristics of all the doctors working in the hospitals including gender, age, experience, education level, title, and monthly income were surveyed. Second, the doctors’ prescribing information was obtained from the information system of the Health Bureau of City Y each month provided by professional information workers. Third, the doctors whose prescriptions were more than 30 before (July to October 2013) and after (November 2013 to February 2014) the intervention were selected to be included in the analysis. Finally, 274 doctors were analyzed in this study, and all the prescriptions of each doctor each month were calculated to reflect each doctor’s prescription behavior.

To ensure quality during data collection, a survey guide was set, and all the researchers were trained according to the guide before the survey. A quality supervisor was also assigned to check the work every day. For the quality assurance of data collection of the doctors’ prescription information, professional information workers were asked to export data according to the format set, and a special computer was used to store the data. All the data used in this study was not freely available and we got the permission of the Health Bureau of City Y.

### Statistical variables

A matched-pair design with PSM was used to analyze the effects of the intervention on the doctors’ prescribing behavior. Three indicators were used: prescription costs, antibiotics use, and injection use. All the three indicators were defined as two categorical variables. If the average expenditure for the prescription of a doctor after the intervention was lower than before, his or her prescription cost was designated as a “decrease”, otherwise (if the expenditure remained the same or was higher), it was designated as a “not decrease”. If the percentage of the prescriptions of a doctor requiring antibiotics after the intervention was lower than before, his or her antibiotics use was defined as a “decrease”, otherwise, a “not decrease”. If the percentage of the prescriptions of a doctor requiring injections after the intervention was lower than before, his or her injection use was defined as a “decrease”, otherwise, a “not decrease”.

### PSM

To balance the covariate difference between the intervention group and the control group doctors, PSM was used to evaluate the effects of intervention by comparing the outcomes in the intervention group with the results in the control group. Control group doctors are suitable matches for intervention group doctors if they have similar observed characteristics as evaluated by a particular distance metric, i.e., the propensity score from a logistic regression model used in this study (Table 1).

**Table 1:** **Description of variables in the logistic model of PSM**

**Variable name**	**Description**
Dependent variable	
Group	intervention group (1); control group (0)
Independent variable	
Gender	male (1); female (0)
Age (year)	continuous
Experience (year)	continuous
Education level	high school or less (1); some college or associates (2); bachelors or higher (3)
Title	not certified (1); certified doctor (2); house physician (3); doctor in charge of a case (4); assistant director physician and above (5)
Monthly income (yuan)	<1500 (1); 1501–2000 (2); 2001–2500 (3); 2501–3000 (4); >3001 (5)

PSM was implemented in two steps: (1) the conditional probability of being highly compensated (i.e., the propensity score) was calculated; and (2) the scores in close proximity to the exposed propensity scores were selected from the control group. Nearest-neighbor matching was performed using a caliper value of 0.03 [14], and caliper matching with one-to-one matches based on the propensity scores was used in this study.

### Data analysis

Independent sample *t*-test or chi-square test was used to analyze the differences of the covariates between the intervention and control groups before and after PSM. *t*-test and chi-square test were used to estimate the association between the intervention and the result indicators, including prescription costs, antibiotics use, and injection use. Statistical significance was accepted at p-value ≤0.05 (SPSS 12.0). The study was approved by the Committee of Tongji Medical College, HuaZhong University of Science and Technology (IORG No: IORG0003571), and the 274 doctors all provided written informed consent.

## Results

### Characteristics of the participating doctors

The average expenditure per prescription of the 274 doctors before the intervention was 42.82 RMB yuan (USD 6.97, the exchange rate was US $1 = RMB 6.14 in March 2014) and after the intervention was 46.68 RMB yuan (USD 7.60). The percentages of prescriptions requiring antibiotics of the 274 doctors before and after the intervention were found to be 63.00% and 61.36%, respectively. The percentages of prescriptions requiring injections of the 274 doctors before and after the intervention were found to be 70.79% and 66.02%, respectively. Among the 274 doctors, 68 doctors (24.82%) reduced the average expenditure per prescription, 123 doctors (44.89%) reduced the percentage of prescriptions requiring antibiotics, and 192 doctors (70.07%) reduced the percentage of prescriptions requiring injections (Table 2).

**Table 2:** **Other characteristics of the investigated doctors**

**Variable name**	**Median ± SD/frequency (percent)**
Gender	
Male	172 (62.77%)
Female	102 (37.23%)
Education level	
High school or less	84 (30.66%)
Some college or associates	139 (50.73%)
Bachelors or higher	51 (18.61%)
Title	
Not certified	12 (4.38%)
Certified doctor	57 (20.80%)
House physician	60 (21.90%)
Doctor in charge of a case	130 (47.45%)
Assistant director physician and above	15 (5.47%)
Monthly income (yuan)	
<1500	63 (22.99%)
1501–2000	101 (36.86%)
2001–2500	62 (22.63%)
2501–3000	130 (47.45%)
>3001	15 (5.47%)
Age (year)	40 ± 9.91
Experience (year)	19.5 ± 12.25

Note: The total number of sampled institutions was 274. For continuous and categorical variables, median ± standard deviation and frequency (percent) were used, respectively.

### Propensity score estimation

The doctors’ propensity scores were estimated using a logistic regression model of the probability of receiving the intervention based on the following: gender, education level, title, income, age, and experience. A total of 108 pairs were obtained after PSM. The utilization of the total sample was 79% (216 out of 274). Table 3 shows the covariate in the pre- and post-matched samples for the intervention and control groups. A significant difference was found between the income of the groups (p < 0.05). After PSM, the difference was insignificant (p > 0.05). Most variables obtained a high p-value after PSM.

**Table 3:** **Variables for per- and post-matched samples for the intervention and control group**

**Variable name**	**Pre-PSM approach**			**Post-PSM approach**		
	**Control group**	**Intervention group**	**P value**	**Control group**	**Intervention group**	**P value**
Gender						
Male	92 (64.34%)	80 (61.07%)	0.576	68 (62.96%)	70 (65.74%)	0.670
Female	51 (35.66%)	51 (38.93%)		40 (37.04%)	38 (34.26%)	
Education level						
High school or less	46 (32.17%)	38 (29.01%)	0.837	37 (34.26%)	34 (31.48%)	0.927
Some college or associates	70 (48.95%)	69 (52.67%)	0.752	50 (46.30%)	54 (50.00%)	0.733
Bachelors or higher (Refer)	27 (18.88%)	24 (18.32%)		21 (19.44%)	20 (18.52%)	
Title						
Not certified	5 (3.50%)	7 (5.34%)	0.199	5 (4.63%)	6 (5.56%)	0.433
Certified doctor	25 (17.48%)	32 (24.43%)	0.123	20 (18.52%)	25 (23.15%)	0.282
House physician	25 (17.48%)	35 (26.72%)	0.090	19 (17.59%)	27 (25.00%)	0.202
Doctor in charge of a case	78 (54.55%)	52 (39.69%)	0.618	56 (51.85%)	45 (41.67%)	0.677
Assistant director physician and above (Refer)	10 (6.99%)	5 (3.82%)		8 (7.41%)	5 (4.63%)	
Monthly income (RMB yuan)						
<1500	30 (20.98%)	33 (25.19%)	0.107	26 (24.07%)	33 (30.56%)	0.636
1501–2000	55 (38.46%)	46 (35.11%)	0.030	39 (36.11%)	41 (37.96%)	0.444
2001–2500	37 (25.87%)	25 (19.08%)	0.015	27 (25.00%)	20 (18.52%)	0.215
2501–3000	16 (11.19%)	13 (9.92%)	0.054	12 (11.11%)	7 (6.48%)	0.163
>3001 (Refer)	5 (3.50%)	14 (10.69%)		4 (3.70%)	7 (6.48%)	
Age (year)	41 ± 9.92	38 ± 9.86	0.055	40 ± 10.74	39 ± 9.43	0.921
Experience (year)	20 ± 13.36	18 ± 10.82	0.088	20 ± 11.62	20 ± 10.19	0.822

Note: For continuous and categorical variables, median ± standard deviation and frequency (percent) were used, respectively. Differences between groups were tested by independent *t* test (Mann–Whitney U test) for continuous variables and by chi-square test for categorical variables. All p-values were two tailed.

### Effect of the intervention on the doctors’ prescribing behavior

Table 4 shows that the average expenditure per prescription, percentage of prescriptions requiring antibiotics, and percentage of prescriptions requiring injections in the baseline were all comparable. After the intervention, a significant difference (p < 0.05) for the percentage of prescriptions requiring injections was found between the intervention and control groups, but this difference did not exist for the indicators of average expenditure per prescription and percentage of prescriptions requiring antibiotics.

**Table 4:** **Effect of the intervention on doctors’ prescribing behavior after intervention (Median ± SD)**

**Variable name**	**Intervention group (N = 108)**	**Control group (N = 108)**	**Z** ^**a**^	**P value**
Average expenditure per prescription of a doctor (RMB yuan)				
	50.07 ± 24.90	43.62 ± 34.41	−2.530	0.110
Percentage of prescriptions requiring antibiotics of a doctor (%)				
	62.49 ± 21.64	60.97 ± 25.86	−0.107	0.915
Percentage of prescriptions requiring injections of a doctor (%)				
	64.66 ± 23.51	70.52 ± 25.57	−2.349	0.019

Note: Non-parametric tests were used because all the variables were not normal; a stands for Mann–Whitney U test.

Table 5 clearly shows the changes in the doctors’ prescription behaviors. After the intervention, 34.26% of the doctors in the intervention group reduced the average expenditure per prescription, whereas 47.22% of the doctors in the control group reduced the average expenditure per prescription. Hence, no significant difference was shown between the two groups (p > 0.05). The ratio of the doctors who decreased the percentage of prescriptions requiring antibiotics in the intervention group was 45.37%, which is higher than that in the control group (41.67%), but no significant difference existed (p > 0.05). Of the doctors in the intervention group, 39.81% reduced the percentage of prescriptions requiring injections, whereas 26.85% of doctors in the control group reduced the percentage of prescriptions requiring injections. These results exhibited a significant difference (p < 0.05).

**Table 5:** **Effect of the intervention on result indicators**

**Variable name**		**Intervention group**	**Control group**	**Total**	**Chi-square value**	**P value**
Prescription costs	Not decrease	71 (65.74%)	57 (52.78%)	128 (59.26%)	3.759	0.053
	Decrease	37 (34.26%)	51 (47.22%)	88 (40.74%)		
	Total	108 (100.00%)	108 (100.00%)	216 (100.00%)		
Antibiotics use	Not decrease	59 (54.63%)	63 (58.33%)	122 (56.48%)	0.301	0.583
	Decrease	49 (45.37%)	45 (41.67%)	94 (43.52%)		
	Total	108 (100.00%)	108 (100.00%)	216 (100.00%)		
Injection use	Not decrease	65 (60.19%)	79 (73.15%)	144 (66.67%)	4.083	0.043
	Decrease	43 (39.81%)	29 (26.85%)	72 (33.33%)		
	Total	108 (100.00%)	108 (100.00%)	216 (100.00%)		

The results from Tables 4 and 5 reveal that after the intervention, more doctors reduced injection use, leading to the lower percentage of prescriptions requiring injections in the intervention group than in the control group.

## Discussion

To the best of our knowledge, this study is the first attempt to evaluate the effect of publicly reporting medicine use information on rational drug use in China. The results show that before the intervention, the average expenditure per prescription of the 274 doctors was 42.82 RMB yuan (USD 6.97), the percentage of prescriptions requiring antibiotics of the 274 doctors was 63.00%, and the percentage of prescriptions requiring injections of the 274 doctors was 70.79%. According to a paper published in health policy and planning (2013) [15], by the end of 2011, the average expenditure per prescription in Hubei Province was 26.67 RMB yuan (USD 4.34) and the percentage of prescriptions requiring injections was 59%. Hence, City Y has higher prescription expenditure and ratio of injection than the average in Hubei Province. However, the World Health Organization (WHO) recommended that the percentage of encounters with antibiotics should range from 20.0% to 26.8%, and the normal range of percent of encounters with injections should be 13.4% to 24.1% [16,17]. Therefore, the drug use in City Y is irrational and needs urgent attention.

In our study, we demonstrated that public reporting of medicine use information has a significant effect on the injection use in the township hospitals of China. Public release of doctors’ performance data can improve doctors’ behavior in prescribing injections. From a policy perspective, our study showed significant implications for the policymakers who were continuously searching for evidence of regulatory measure. Making doctors’ and hospitals’ information on injection use open would improve the doctors’ prescription behavior and promote rational drug use. However, the mechanism on how public reporting of medicine use information affected injection use is not included in this paper and thus requires further studies.

Although Berwick thought that the effect came from the selection of consumers and the self-effort of providers to improve [4], certain barriers may still exist. Certain researchers believe that the selection of consumer pathway may be interrupted by the low level of awareness about the information [18-20]. For example, a research found that only 12% of patients consider the information released [21]. In our study, best effort was exerted to disseminate the information in an appropriate format and manner that was understandable and would motivate the patients to act on the information; however, the patients’ comprehension may be insufficient, which became a barrier to consumers’ selection [22,23]. Other researchers believe that to provide the best chance for success, the release of information should follow certain criteria such as accuracy, relevance, timeliness, cost-effectiveness, and accountability [24]. Therefore, in future studies, evaluating the format or way of information disclosure may be done. Although we tried to communicate and convey medicine use information more effectively to patients, patients might have little or no choice but to go to the nearest hospital for reasons of district and policy issues in China. Furthermore, the incentive to sustain the effort of rational drug use can also affect doctor behavior [10]. In our study, we reported three indicators to patients and doctors, but we only saw the effect on injection use. Certain researchers found that the effect of disclosure on quality depended on the type of services [25,26], and in our research, the results indicated the effect of disclosure on medicine use might depend on different indicators, which still need further research. These indicators might be the reasons why we did not see the effects of public reporting of medicine use information on prescription costs and antibiotics use.

From a methodological perspective, we have done a quasi-experimental design to eliminate the difference of institutions and used PSM to avoid the risks of selection bias based on doctors’ characteristics in analyzing the effect of public reporting of medicine use information on rational drug use. By using logistic regression, the propensity score was estimated with the intervention as the outcome and the doctors’ characteristics as the predictive variables. A total of 108 pairs of doctors shared close propensity scores, and similar characteristics were included in our study. The uniformity in the measured risk factor distribution indicates that the distribution of unmeasured variables is balanced, although the propensity scores cannot remove hidden biases except to the extent that the unmeasured prognostic variables are correlated with the measured covariates used to compute the score. Therefore, efficient development of health care policies can be supported using propensity score analysis [27].

Our study also has certain limitations. First, the data analyzed in our study were obtained four months before the intervention and four months after the intervention; hence, long-term effect was not discussed. However, we believe that understanding the immediate effect of the intervention is also important and could provide useful implications. Second, we did not discuss the responses of both patients and doctors, which would better explain the findings in our research and needs further research. Third, we did not evaluate the format or way of information release. In addition, we did not include variables of the patients in our analysis. Even though some researchers used certain criteria, no indicator system has been reported, indicating that much work still needs to been done in this area.

## Conclusions

Irrational use of drug is still a policy issue in township hospitals in our study area. This study is the first attempt to evaluate the effect of public reporting of medicine use information on rational drug use in China. After controlling the difference of hospitals’ characteristics in designing and using PSM to partially adjust the selection bias of doctors’ characteristics, we demonstrated that public reporting of medicine information could decrease the percentage of prescriptions requiring injections in township hospitals of China. However, this effect was not observed on prescription costs and antibiotics use. The findings enrich the evidence of health care transparency on quality and provide references for China and other countries to promote health care transparency. Analyses of the mechanism and long-term effect of public reporting of medicine use information are recommended for future studies.
